# Exercising With Embodied Young Avatars: How Young vs. Older Avatars in Virtual Reality Affect Perceived Exertion and Physical Activity Among Male and Female Elderly Individuals

**DOI:** 10.3389/fpsyg.2021.693545

**Published:** 2021-10-25

**Authors:** Jih-Hsuan Tammy Lin, Dai-Yun Wu

**Affiliations:** ^1^Department of Advertising, College of Communication, National Chengchi University, Taipei, Taiwan; ^2^Taiwan Institute for Governance and Communication Research, Taipei, Taiwan; ^3^Department of Communication and Technology, National Yang Ming Chiao Tung University, Hsinchu, Taiwan

**Keywords:** Proteus effect, elder exercise, avatar, embodiment, perceived exertion, physical activity, virtual reality exercise, sex difference

## Abstract

This study demonstrated that implementation of the Proteus effect via manipulation of avatar age in VR is effective among elderly individuals in the context of exercise. One hundred and four elderly adults aged 60 years and older who did not engage in vigorous physical activities participated in this experiment with a 2 (avatar age: young vs. older) × 2 (sex: male vs. female) design. The results showed that the embodiment of younger avatars (age approximately 20 years) in VR leads to greater perceived exercise exertion regardless of sex after controlling for age and emotion. Older adults with young avatars perceived a greater contribution of efforts to exercise. This study also found that among those who did not engage in vigorous exercise, female older adults who embodied young avatars reported greater self-efficacy for future exercise and greater physical activity during the exercise phase than those who embodied older avatars. This study suggests that females are more likely to be motivated to continue exercising through young avatar embodiment. In contrast, female elderly who embodied old avatars reported significant fewer physical activity than male elderly who embodied old avatars. This indicated that the Proteus effect had stronger effects among females than among older males. Although we found the Proteus effect through VR avatar manipulation, the effect was temporary and limited to the experimental phase. This study is the first to examine the Proteus effect among elderly individuals in the context of exercise. It also contributes to the literature by indicating that avatar age manipulation is an effective means of promoting exercise among elderly individuals and helping them achieve exercise outcomes. This study further demonstrates that female elderly individuals respond to young avatars differently than male elderly individuals, with female elderly individuals showing more positive effects of young avatar embodiment than males. Implications and theoretical contributions are discussed.

## Introduction

Using communication technology to encourage older adults to engage in safe and moderate exercise is an important goal of scholars and society (Kim et al., 2013). According to the World Health Organization (World Health Organization [WHO], 2020), older adults aged 65 years old and above should engage in at least 150 min of moderate-intensity aerobic physical activity per week. Existing video game-based interventions for elderly individuals have indicated positive results and responses among elderly participants (Cyarto et al., 2016). Specifically, compared to video-streamed channels, avatar manipulations and representation in exergames (e.g., video games promoting exercise) have shown promising outcomes for elderly individuals and offer greater privacy (Cyarto et al., 2016). With the commercialization of virtual reality (VR) systems and stand-alone VR machines, VR has garnered considerable attention from both the exergame industry and scholars for promoting exercise. Several VR training programs have shown positive results, such as in improving elderly individuals’ balance, posture and muscle strength through VR exercise intervention (Kim et al., 2013; Lee et al., 2017; Monteiro-Junior et al., 2017). However, the potential effects and underlying mechanism of avatar embodiment manipulation in VR exercise among elderly individuals remain unknown and require investigation.

Embodiment manipulation in VR has received increasing attention in the past decade (Ratan et al., 2019), with scholars exploring how changing one’s embodied avatar with various traits can influence an individual’s attitude and behavior (Yee and Bailenson, 2007; Yee et al., 2009). Yee and Bailenson (2007) theorized the Proteus effect as a top-down effect in which the appearance and traits of an embodied avatar in VR influence one’s self-perception and behavior. For example, participants embodying avatars with attractive faces engaged in more self-disclosure and maintained a shorter physical distance from others while talking to others in VR, and participants embodying taller avatars were more likely to turn down unfair money split offers than those embodying shorter avatars (Yee and Bailenson, 2007). Compared to elderly individuals who embodied adult avatars, elderly individuals who embodied avatars who appeared to be 4 years old perceived themselves to be younger and were more likely to overestimate object sizes (Tajadura-Jiménez et al., 2017). Evidence has shown that people’s perceptions of their body are malleable and depend on sensory cues in VR (Tajadura-Jiménez et al., 2017; Tacikowski et al., 2020).

One specific stream of avatar embodiment research has focused on exercise. However, most studies have employed video game-based avatars shown on a screen where the participants can see their own actual bodies while exercising (Li et al., 2014; Waddell et al., 2015; Peña et al., 2016; Joo and Kim, 2017). Very few studies (Fox and Bailenson, 2009; Reinhard et al., 2020) have explored embodiment illusion in VR. In addition, to the best of our knowledge, avatar manipulation in VR has not been applied to elderly individuals in the context of exercise. In this study, we manipulated the age of the embodied avatars so that elderly individuals embodied either older avatars or younger avatars (i.e., 20 years old), and we examined their self-efficacy and physical activity in VR. We also investigated the underlying theoretical mechanism of avatar manipulation in VR. Last, we explored potential sex differences in the persuasion effects of embodiment manipulation on exercise since females are more likely to internalize idealized thin body representations in the media (e.g., Hargreaves and Tiggemann, 2003; Levine and Murnen, 2009). In this paper, we first discuss the main outcomes expected from this study, then review the previous literature on the avatar manipulation approach, and then end with a discussion of the theoretical mechanisms of the Proteus effect and self-concept.

### Exercise-Related Outcomes

Regular weekly aerobic exercise is recommended for older adults for healthier physical and mental well-being and to reduce potential health risks (World Health Organization [WHO], 2020). Among several interventions designed to encourage elderly individuals’ physical activity, self-efficacy for future exercise is a central goal and is the most important variable among the psychological factors related to physical activity (Anderson et al., 2006). As a core construct in social cognitive theory (Bandura, 1977), self-efficacy is defined as “the belief in one’s capabilities to organize and execute the courses of action required to produce given attainments” (Bandura, 1977, p. 3). Self-efficacy for exercise thus describes one’s belief that he or she is capable of engaging in future exercise or physical activity. Research has indicated a positive association between self-efficacy and regular exercise among elderly individuals (Hwang and Chung, 2008). Self-efficacy for exercise among elderly individuals was even found to be more predictive of long-term exercise (7–12 months) than exercise-related social support in intervention-based programs (Brassington et al., 2002).

Another common goal is exercise as a behavioral outcome among elderly people, i.e., their actual physical activity. Defined as a “bodily movement produced by skeletal muscles that requires energy expenditure,” physical activity “refers to all movement, including during leisure time, for transport to and from places or as part of a person’s work” (World Health Organization [WHO], 2020). The WHO recommends various levels of moderate- and vigorous-intensity physical activity for improving health. In sum, physical activity refers to all physical movements made through various types of exercise such as walking, running, or swimming. Greater physical activity refers to higher degrees of physical movement. Among sensor-based physical activity monitoring systems, accelerometers have been reported to be effective and reliable in measuring physical activity among elderly individuals (van Schooten et al., 2015). Accelerometer sensors assess physical activity based on the vector magnitude (created by movements on the three axes) and step counts, which predict energy expenditure. That is, accelerometer sensors record the movements a participant makes in horizontal (*x*-axis), vertical (*y*-axis), forward, and backward (*z*-axis) directions, which created the vector magnitude representing physical movements during exercise. Accelerometer sensors also count the number of steps a participant takes during the assessment period. Exergame research (Peng et al., 2015) assesses accelerometer data upon engagement in physical activity, with higher values of vector data representing greater engagement in activities and physical movement.

Furthermore, not only physical activity intention and behavior but also one’s effort devoted to exercise is important because some programs require precise movements for rehabilitation or further efforts for the advanced training of specific muscles. Borg’s rating of perceived exertion (RPE) refers to the level of perceived effort in an exercise based on the estimation of “effort and exertion, breathlessness, and fatigue during physical work” (Borg, 1998, p. v). A higher RPE indicates more intensive exercise requiring higher effort. RPE measures subjective perception of self-effort during the exercise. Higher effort devoted to exercise can indicate greater engagement or higher requirement of the physical task.

### Avatar-Based Exercise Interventions

Academics have examined the effects of the traits of digital avatars in exergame programs on players’ exercise outcomes and attitudes (Li et al., 2014; Joo and Kim, 2017). Most exergames are video game-based programs using Nintendo Wii and Xbox motion-sensing systems, which allow participants to see their avatars on TV screens and simultaneously see their actual bodies while engaging in exercise. The results of these studies showed that the provision of reinforcement through this self-modeling approach encouraged participants to exercise harder. For example, participants with normal-weight avatars had better exercise outcomes, such as exercise attitude, motivation and game performance, than those with overweight avatars (Li et al., 2014). In addition, compared to participants with overweight avatars, for those with normal-weight avatars, there was a greater association between the avatar’s healthy behavior and the participant’s step counts (Joo and Kim, 2017). Even without any avatar manipulation, simply using an avatar in Wii boxing significantly reduced social physique anxiety and increased enjoyment among participants with high body dissatisfaction compared with participants with low body dissatisfaction (Song et al., 2014).

Social dynamics have also been explored (Peña et al., 2016) in the context of video game-based exercise examination using avatar manipulation. In addition to the main effect of the use of normal-weight avatars leading to greater physical activity among male participants than the use of normal-weight avatars, the body sizes of participant and opponent avatars also influenced participants’ exercise behavior. Participants engaged in less physical activity when they perceived the opponent avatars to be more obese than their own avatars. Participants also engaged in less physical activity when they perceived their own avatars to be more obese than the opponent avatars.

Scholars have explored avatar manipulation in VR. In the context of exercise, Fox and Bailenson (2009) asked the participants to view their virtual bodies from a third-person perspective, and the results indicated that viewing a virtual body with reinforcement (i.e., gained or lost weight) affected their own exercise behavior—the reinforcement group engaged in more voluntary exercise. In the second study, they found that viewing virtual bodies with models of their own heads made the participants exercise more than viewing virtual bodies with other people’s heads. In the third study, the participants engaged in more exercise in the following 24 h when they viewed a self-avatar running on a treadmill in VR than when they viewed other avatars running or a self-avatar loitering. These results indicated that self-referencing in VR using avatar manipulation has both temporary and prolonged effects on exercise behavior.

Another form of avatar manipulation in VR is embodiment, in which participants directly embody the virtual body as opposed to seeing an avatar on the screen. When embodying a virtual body, a participant can look down and see his or her body as the virtual body and look at his or her virtual body in the mirror as in reality. Seeing is different from being (Yee and Bailenson, 2009), which supports the concept of the Proteus effect (Yee and Bailenson, 2007), in which the traits and appearance of embodied avatars change participants’ actual behavior. One study (Reinhard et al., 2020) tested the age difference (i.e., younger vs. older) in walking speed among undergraduate students. The results showed that participants who had embodied an older avatar in a previous test walked significantly slower than those who had previously embodied younger avatars. They also indicated fast decay effects of the Proteus effect on participants. Furthermore, only two-thirds of participants who had high spatial presence showed such effects. Reinhard et al. (2020) indicated that perceiving themselves as old decreased young participants’ walking speed, and only high levels of spatial presence led to this effect.

Regarding VR-based exercise programs targeting elderly individuals, the existing research has focused on designing programs tailored to elderly individuals with various symptoms or rehabilitation needs. For example, in a 6-week VR exercise program for elderly individuals over 65 years old (Lee et al., 2017), those who participated in the intervention demonstrated significantly improved static and dynamic balance from baseline to posttest, while the control group did not. To the best of our knowledge, no research has tested the effects of avatar manipulation among elderly individuals, and the effects and underlying mechanisms remain unexplored.

### Underlying Mechanism: Proteus Effect and Self-Concept

Most research has attributed the effects of avatar manipulation on participants’ behavior to the Proteus effect (Yee and Bailenson, 2006). A meta-analysis of 46 quantitative experimental studies (Ratan et al., 2019) showed small-to-medium effect sizes, with the participants conforming their behavior to the characteristics of avatars assigned to them. For example, Yee et al. (2009) found that avatar height and attractiveness both affected players’ performance in an online game. In the online game World of Warcraft, taller avatars, and more attractive avatars showed better game performance than shorter and unattractive avatars. In a subsequent study (Yee et al., 2009), the authors found that the Proteus effect induced in the virtual world transferred to later face-to-face interactions. Participants who embodied taller avatars in a virtual environment later negotiated more aggressively in a face-to-face context than those who embodied shorter avatars.

Yee and Bailenson (2006) explained that embodiment changes participants’ self-perception in VR because people rely on external cues, such as the traits of avatars, to form their selves. Therefore, the traits and characteristics of the embodied avatars change participants’ attitudes and behavioral conformity. Peña et al. (2009) theorized that this phenomenon is based a priming mechanism by which limited cues in virtual environments prime participants’ attitudes toward and expectations of such behavioral cues. Ratan and Dawson (2016) theorized this phenomenon to occur through schema activation; they suggested that when embodying an avatar, participants activate schemas related to self that are closely connected to traits of the avatar. In a virtual environment, when participants engage in a certain activity with assigned avatars, they then develop self-perceptions to plan how they should act.

The above theoretical arguments implicitly suggest the association between the self and the avatar as the character that one identifies with in digital games (Klimmt et al., 2009). Klimmt et al. (2009) theorized character identification to be a temporarily altered self-concept, as one temporarily adopts the character’s traits when using the avatar to play a game. Tajadura-Jiménez et al. (2017) examined the self-concept as the underlying mechanism for avatar manipulation in VR. For example, older women who embodied a 4-year-old girl avatar perceived their own self-concept to be younger than did those who embodied a 60-year-old woman avatar. This study adopted the altered self-concept as the potential mechanism. In the exercise context, older adults who embody a young avatar should have a younger self-concept and better exercise outcomes than those who embody an older avatar. When such individuals perceive themselves as young, they may be willing to devote more energy to exercise, resulting in more movement. In the context of aerobics, being in poor physical condition may result in making fewer physical movements and primarily smaller, shorter-range movements. In contrast, being in better physical condition allows participants to fully execute aerobic movements. Therefore, perceiving oneself as young equates to better physical fitness, which further leads to more physical activity. In addition, those with this perception should exhibit greater self-efficacy to engage such exercise in the future. We therefore define better exercise outcomes as higher levels of physical activity and higher degrees of self-efficacy to engage in future exercise.

**H1:** Elderly individuals embodying young avatars will demonstrate (a) greater self-efficacy for future exercise and (b) greater physical activity than elderly individuals embodying older avatars.

**H2:** Elderly individuals embodying young avatars will perceive themselves to be younger than those embodying older avatars.

Regarding perceived exertion, existing research on the Proteus effect (Kocur et al., 2020) has found that those who embodied a muscular avatar reported lower perceived exertion when lifting weights than those who embodied a normal body avatar. In addition, even when holding the same weight, those who embodied the normal body avatar perceived the task as more demanding than those who embodied the muscular avatar. For the elderly, embodying a younger body may alter their self-concept of being young, and they may perceive the exercise as effortless and report lower perceived exertion. However, it is also possible that elders embodying a young avatar are more motivated to distribute more efforts to the task. In the context of aerobics, better exercise outcomes should include devoting more energy to engaging in movement, and thus greater perceived exertion is an ideal outcome. Perceiving themselves as young leads participants to devote energy more to the assigned movements, leading to higher levels of perceived exertion. Perceived exertion is closely tied to age, as age represents one’s physical condition. Older adults perceive the same task as more demanding and thus requiring greater exertion (Allman and Rice, 2003). In addition, VR technology may introduce novel effects in which exercise in a virtual environment results in greater exertion, energy, and enjoyment than in real-world exercise (Chuang et al., 2003; Plante et al., 2003; Fox and Bailenson, 2009). Therefore, regarding perceived exertion, we included age and emotion (i.e., affect and arousal) as control variables. Furthermore, self-concept is theorized as the underlying mechanism in this study by which avatar age leads to altered self-concept, resulting in greater exercise outcomes. Therefore, we propose the following research question and mediation hypothesis.

**RQ1:** How do elderly individuals embodying young avatars report their perceived exertion compared to those embodying older avatars after controlling for age and emotion?

**H3:** Self-concept will mediate the effects of avatar age on exercise outcomes: (a) greater self-efficacy for future exercise and (b) greater physical activity.

**RQ2:** How does self-concept mediate the effects of avatar age on perceived exertion, controlling for age and emotion?

Sex differences have rarely been explored in avatar manipulation and almost never in the context of exercise among elderly individuals. The existing literature on exercise has either focused on individuals of a single sex (Peña and Kim, 2014; Peña et al., 2016) or included individuals of both sexes but rarely explored sex differences. The literature on regular exercise interventions without technological components showed sex differences in aerobic exercise efficacy (Barha et al., 2017). Female participants who participated in the intervention showed improved aerobic exercise efficacy and cognition compared to that of participants who received usual care plus education, and this effect was retained at follow-up. However, such an effect was not observed among male participants. Since females are more likely to experience body-related schema activation from viewing thin-ideal images than male participants (Hargreaves and Tiggemann, 2003), it is likely that female participants would have better exercise outcomes when embodying younger avatars. However, the potential moderating effect of sex on the association of avatar manipulation with exercise outcomes in VR exercise has not been explored. We thus posed the following research question:

**RQ3:** Does sex moderate the association of avatar age with (a) perceived exertion, (b) self-efficacy for future exercise, (c) physical activity, and (d) age self-concept?

We examined the above hypotheses and research question during an exercise phase and a voluntary exercise phase when the participants embodied their assigned avatars. We also explored engagement in physical activity and self-efficacy the day after the experiment in the follow-up phase.

## Materials and Methods

### Design and Participants

The experiment used a 2 (avatar age: young vs. older) × 2 (sex: male vs. female) between-subjects design. The participants embodied an older or a younger avatar of the same sex as themselves and exercised following along with a video in a virtual gym. In addition, since this study aims to motivate elderly individuals to engage in more exercise, we focused on recruiting those who aged 60 or older (United Nations, 2019) and recruiting those who did not engage in vigorous physical activity. According to the American Heart Association (2018), vigorous physical activity involves considerable exertion through activities such as running, aerobic dancing, lap swimming, tennis, jumping rope, and cycling 10 miles per hour or faster. Those reporting engaging in 0 min of vigorous exercise were chosen for this study.

The participants were recruited in three ways. First, recruitment invitations were sent via a campus announcement email to all staff at a national university in northern Taiwan. Second, recruitment was conducted in community centers near the university. Third, with the assistance of the District Health Center, recruitment information was handed out by community nurses during home visits to elderly individuals. Individuals who were aged 60 and over, were not cognitively impaired and did not require a walking stick or walking frame were invited to join this study. In total, 121 people were recruited for the study. After selecting those who did not engage in vigorous exercise, 104 elders participated in the experiment (age range: 60–88 years, *M* = 70.39, *SD* = 6.51; 26 female and 25 male participants embodying older avatars and 27 female and 26 male participants embodying young avatars). The participants were stratified by sex and randomly assigned to either the young avatar group or the older avatar group.

### Stimulus

**FIGURE 1:**
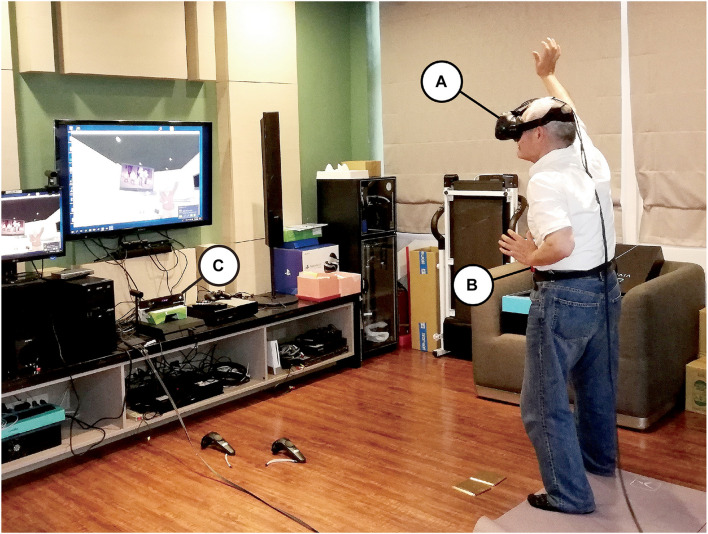
Experimental setup. **(A)** hTC VIVE head-mounted display. **(B)** ActiGraph wGT3X-BT accelerometer. **(C)** Microsoft Xbox One Kinect Sensor.

**FIGURE 2:**
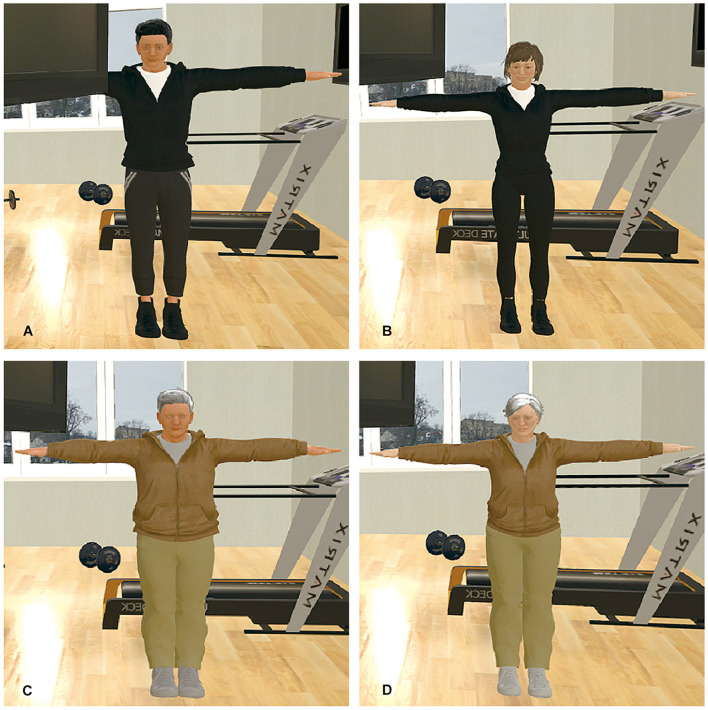
Avatars. **(A)** Young male. **(B)** Young female. **(C)** Older male. **(D)** Older female.

**FIGURE 3:**
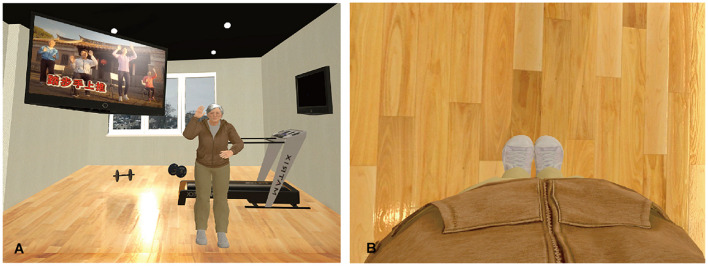
The environment of the virtual gym. **(A)** Overview of the virtual environment for the elderly self-avatar in the exercise phase. **(B)** Participants could see their virtual body from a first-person perspective.

The participants viewed the virtual world using an hTC VIVE head-mounted display (HMD). A 3 × 2.5-m space was established for this experiment, and the participants could walk freely inside the preset space (Figure 1). The virtual scene began with the participant standing in a gym facing a mirror wall, and the body of the participant was replaced with a virtual body of the same sex as himself or herself. The virtual environment was built using Unity game engine.^1^ The participants’ actual body positions and movements were tracked using a Microsoft Xbox One Kinect Sensor and were mapped onto the virtual body so that the virtual body moved in accordance with the movements of the participant’s actual body in real-time. The avatars were created using MakeHuman,^2^ an open-source tool for 3D character creation. Four avatars were created: a young male, a young female, an older male, and an older female (Figure 2). The self-avatar of the same sex (either the young or the older avatar) was embodied, and the participants could see the virtual body of their self-avatars from a first-person perspective or in the mirror in front of them. A virtual television screen was hung on the upper left side of the mirror wall and played a workout video for seniors so that the participants could check their actions in the mirror while watching the video on TV (Figure 3).

### Procedure

All participants completed a pre-intervention questionnaire that collected demographic information and asked questions about their exercise behaviors the week before they were invited to the laboratory. Upon arriving at the laboratory, the participants signed the consent form, which informed them that they would participate in a VR experience. The research team helped the participants place an ActiGraph wGT3X-BT accelerometer unit on their waist to record their continuous physical activity (Figure 1B). Then, they were assisted in putting on the VR goggles and were instructed to follow the prerecorded voice instructions in the virtual scene.

**FIGURE 4:**
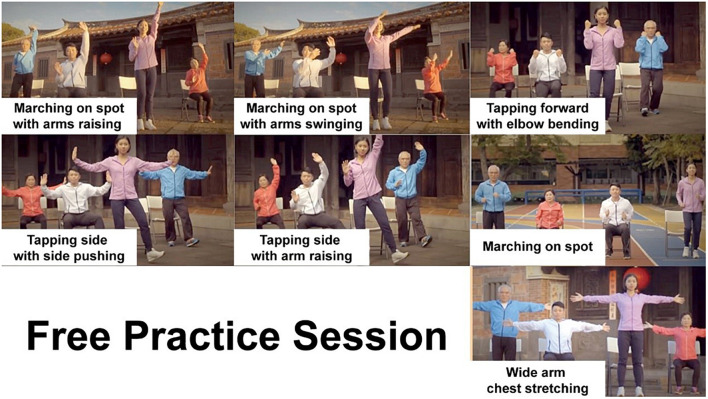
The exercises featured in the video.

During experimental phase one (1 min), the participants entered a virtual gym and saw themselves facing a large mirror wall. The voice instructions guided the participants to become familiar with the surroundings and their virtual bodies by looking around, looking down and looking in the mirror. Then, they were instructed to perform a series of simple movements, such as knee lifts and arm raises, to help them associate their actual bodies’ physical movements with their avatars’ movements. During phase two, a workout video played on the virtual TV, and the participants were asked to exercise following the video. The video featured 7 simple exercises, such as marching in place, tap out, heel down in the front, and chest stretch (Figure 4). The length of the video was 4 min and 30 s. In phase three, the participants were told that there was still some extra time and that they could keep exercising, practice the movement in the video freely in front of the mirror or hang out in the virtual environment until the time was up. The voluntary phase lasted 3 min. After the voluntary exercise phase, the research team helped the participants take off the VR goggles and ActiGraph wGT3X-BT accelerometer and asked the participants to assess their perceived level of exertion. Then, the participants were asked to sit in front of a computer and complete an Implicit Association Test (IAT) and Affect Grid in order and then complete a questionnaire on their self-concept, self-efficacy regarding engaging in exercise and demographic information.

The follow-up phase was conducted the next evening after the participant left the laboratory. The research team called the participants to ask about their engagement in physical activity that day and their self-efficacy for future exercise.

### Measurements

*Perceived exertion* (*M* = 11.66, *SD* = 2.72) was assessed using the Borg Rating of Perceived Exertion Scale (Borg, 1998). This scale ranges from 6 (no exertion at all) to 20 (maximal exertion). The participants were asked to select a number from the scale that best represented their exertion during the physical activity based on their overall feelings of effort and fatigue.

*Self-efficacy for exercise* (α = 0.86, *M* = 6.04, *SD* = 0.85) was measured using a four-item scale modified from Lorig et al. (1996). The participants rated their degree of agreement (1 = strongly disagree and 7 = strongly agree) with statements such as “After the VR experience, I am confident that I can do some simple daily workout” and “In the next six months, I am confident that I can do aerobics regularly.”

*Physical activity* was measured by the ActiGraph wGT3X-BT accelerometer, which collected vector magnitude of the body movements (experimental phase, *M* = 3948.84, *SD* = 1936.08; voluntary phase, *M* = 2747.52, *SD* = 2271.10) and step counts (experimental phase, *M* = 33.03, *SD* = 27.06; voluntary phase, *M* = 25.45, *SD* = 42.49). Please note that these are the sum of all vector movements and step counts during the phases. The device records the acceleration on the three axes, and the raw data is summed into chunks of data called “epochs” and the *G*-values are converted to activity counts (ActiGraph Corporation, 2018). The term “counts” does not mean that the vector magnitude data is also generated by counting (ActiGraph Corporation, 2008). Rather, “Activity that caused the acceleration signal to exceed the threshold was ‘counted’ as activity; anything below this threshold was ignored. At the end of the measurement period, the number of activity ‘counts’ would be recorded” (ActiGraph Corporation, 2008). The vector magnitude was the Euclidean vector of the three axes, that is, the square root of the sum of the squares of all three axes. Step counts represent one participant’s steps made during the experimental period. We collected data at 100 Hz, which means there would be 100 samples per second. The data was exported to a CSV file using the official software, ActiLife.^3^ The exporting epoch was set to 10 s, which means the software will sum the acceleration data and produce one data point every 10 s. All the epochs during the measurement time were added up to represent the total activity during the phase (e.g., 4.5 min for the experimental phase, and 3 min for the voluntary practice phase). Higher numbers of the sum of the vector magnitude counts indicates greater body movements. Taking all indicators together, we can determine whether the participants engage in more physical movements based on these data (Lin, 2015; Peng et al., 2015).

*Self-concept* was assessed using both explicit and implicit methods. The explicit self-concept (α = 0.94, *M* = 4.04, *SD* = 1.44) was measured using a four-item semantic differential scale developed by this study. The participants were asked to answer how they would describe themselves using a 7-point scale between two polar adjectives (young/old related adjectives written in Chinese). We also employed IAT to measure the implicit beliefs that the participants may be unable to report (Greenwald et al., 1998). This test has been widely used for the assessment of self-concept (Schnabel et al., 2008; Suslow et al., 2014). Participants are asked to quickly sort words into categories on the left and right sides of the computer screen using two response keys on the keyboard. The self-concept is assessed based on how long it takes a person to sort the words. Shorter response times indicate stronger associations between concepts assigned to the same key.

The IAT has been successfully used to measure the alternation of self-concept resulting from avatar embodying or game character identification experiences. For instance, Klimmt et al. (2010) used the IAT to demonstrate that after playing a military first-person shooter, players tend to associate themselves with military-related concepts. Banakou et al. (2013) employed the IAT to assess the self-concept changes of participants who embody a child or adult avatar. Banakou et al. (2013) demonstrated that participants in the child avatar group reacted significantly faster to classifications of the self with child-like rather than adult-like attributes. Starr et al. (2019) also employed the IAT to examine the effects of a first-person perspective virtual reality experience on participants’ self-concept in relation to physical science, technology, engineering, and mathematics (pSTEM). Based on the literature mentioned above, the IAT was considered an ideal tool for assessing implicit changes in self-concept through the mediated experiences.

Followed the procedure in Greenwald et al. (1998), the IAT in this study consisted of five blocks. In the first and second blocks, the participants practiced a target concept discrimination task by categorizing words into “self” or “others” categories and an attribute discrimination task by categorizing words into “young” or “older” categories separately. In the third block, the participants sorted words into two combined categories, each including one target and one attribute concept that were assigned to the same key in the preceding two steps (e.g., “self” and “older” shared the left key, and “others” and “young” shared the right key). In the fourth block, the participants practiced another attribute discrimination task with reversed key assignments. The fifth block was similar to the third, but the concepts that shared the same keys were switched (e.g., “self” and “young” shared the left key, and “others” and “older” shared the right key). The orders of blocks 2–3 and blocks 4–5 were counterbalanced to prevent possible effects caused by order. The IAT score (*M* = −390.36, *SD* = 409.73) was computed by subtracting the mean response time for the block in which others and young shared the same key and self and older shared the same key (Step 3) from the block in which self and young shared one key and others and older shared another key (Step 5). Subjects whose error rate was higher than 20% were dropped in the IAT analysis, response times greater than 3,000 ms were recoded as 3,000 ms, and those less than 300 ms were recoded as 300 ms before computing. We considered participants to perceive themselves as younger rather than older if they more quickly categorized words when “self” and “youthful” shared a response key and when “others” and “aged” shared a response key than when the opposite was true.

*Emotion* was assessed using the Affect Grid (Russell et al., 1989), which is a means of measuring human emotions along the dimensions of valence (pleasant—unpleasant) and arousal (high arousal—sleepiness) using a 9 × 9 grid. Participants were asked to rate their current emotional state and place a checkmark somewhere in the grid to indicate this state [pleasant (*M* = 6.22, *SD* = 1.06) and arousal (*M* = 6.03, *SD* = 1.15)].

*Age* (Range: 60–88; *M* = 70.39, *SD* = 6.51) was assessed by inquiring the participants’ birth years. In Taiwan, the elderly frequently lose track of their age, but most can remember their birth year correctly. Therefore, we asked participants about their birth year and calculated their current age. The participants vary enough in age for us to use age as a covariate.

*Next-day exercise* (*M* = 37.72, *SD* = 52.73) was assessed by asking the participants which physical activities they engaged in that day and the duration of the activities. The amount of time the participants spent on moderate and high-intensity exercise were added and calculated (in minutes). *Next-day self-efficacy for exercise* (α = 0.89, *M* = 5.84, *SD* = 1.05) was measured using the same scale as that used after the intervention.

## Results

**FIGURE 5:**
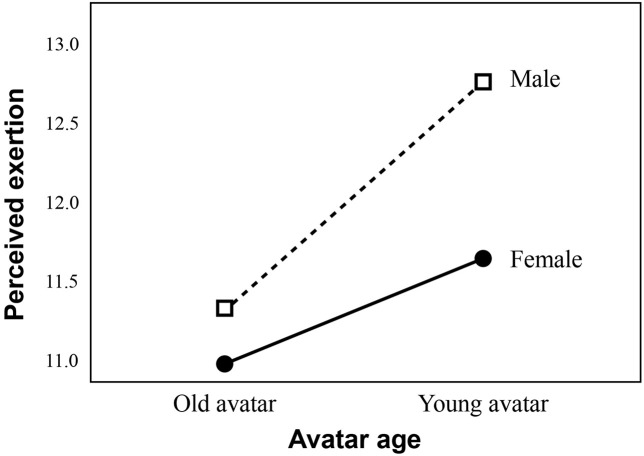
The main effect of avatar age on perceived exertion during the exercise phase (controlling for age and emotion).

The descriptive statistics and correlations of all of the study variables are listed in Table 1. This study employed a 2 (avatar age: young vs. older) × 2 (sex: male vs. female) factorial design. We first examined the perceived exertion outcome **(RQ1)** during the exercise phase. Levene’s test was not significant (*p* = 0.49); thus, no adjustment was adopted for the following analysis. However, Bonferroni adjustment was employed because we included covariates in this analysis. The 2 × 2 ANCOVA controlling for age and emotion (i.e., pleasant and arousal) indicated a significant main effect of avatar age on perceived exertion (Figure 5), *F* = 4.14 (1, 97), *p* = 0.045, partial η^2^ = 0.041. Regardless of sex, the participants who embodied young avatars reported higher perceived exertion (adjusted *M* = 12.19, *SE* = 0.37, *N* = 53) than those who embodied older avatars (adjusted *M* = 11.13, *SE* = 0.37, *N* = 51). We conducted a simple main effect analysis and found that among older male adults, the young avatar group (raw *M* = 12.77, *SD* = 2.86, *N* = 26; adjusted *M* = 12.76; *SE* = 0.52) reported higher levels of perceived exertion than the older avatar group (raw *M* = 11.2, *SD* = 2.55, *N* = 25; adjusted *M* = 11.297; *SE* = 0.539), *F* = 4.49 (1, 97), *p* = 0.036. Among the female participants, there was no significant difference in the perceived exertion of the female participants who embodied young (raw *M* = 11.48, *SD* = 2.83, *N* = 27; adjusted *M* = 11.63; *SE* = 0.516) vs. old (raw *M* = 11.19, *SD* = 2.25, *N* = 26; adjusted *M* = 10.956; *SE* = 0.549) avatars, *F* = 0.16 (1, 97), *p* = 0.69. Therefore, the main effect of avatar age on perceived exertion mainly came from male participants. The main effect of sex, *F* = 1.93 (1, 97), *p* = 0.17, partial η^2^ = 0.02, and the interaction effect of avatar age with sex **(RQ3a)**, *F* = 0.51 (1, 97), *p* = 0.48, partial η^2^ = 0.005, were not significant.

**TABLE 1:** Descriptive statistics and correlations of study variables.

		**1**	**2**	**3**	**4**	**5**	**6**	**7**	**8**	**9**	**10**	**11**	**12**
1	RPE	1											
2	SE	–0.154	1										
3	IAT score	–0.006	0.179	1									
4	ESC	0.093	−0.228*	0.022	1								
5	Steps-E	–0.118	0.316**	–0.048	−0.198*	1							
6	VM-E	0.156	0.169	–0.008	–0.038	0.623**	1						
7	Steps-V	–0.108	0.210*	–0.022	−0.225*	0.377**	0.109	1					
8	VM-V	0.018	0.141	0.178	–0.142	0.379**	0.396**	0.703**	1				
9	PL	–0.041	0.332**	–0.131	–0.130	0.071	0.051	0.132	0.157	1			
10	AR	–0.152	0.453**	–0.157	–0.109	0.274**	0.207*	0.157	0.152	0.646**	1		
	Next-day variables												
11	SE-N	0.112	0.623**	–0.131	–0.117	0.171	0.111	–0.041	–0.057	0.294**	0.293**	1	
12	EX-N	0.075	0.186	–0.134	–0.082	0.209*	0.071	0.152	0.010	0.187	0.127	0.270**	1

	*Mean*	11.66	6.04	–342.00	4.14	33.03	3948.84	25.45	2747.52	6.22	6.03	5.84	37.72
	*SD*	2.72	0.85	447.06	1.51	27.06	1936.08	42.49	2271.10	1.06	1.15	1.05	52.73

RPE, Perceived exertion; SE, Self-efficacy; IAT score, Implicit self-concept; ESC, Explicit self-concept; Steps-E, Step counts during the exercise phase; VM-E, Vector magnitude during the exercise phase; Steps-V, Step counts during the voluntary phase; VM-V, Vector magnitude during the voluntary phase; SE-N, Next-day self-efficacy; EX-N, Next-day exercise. *p < 0.05, **p < 0.01.

**FIGURE 6:**
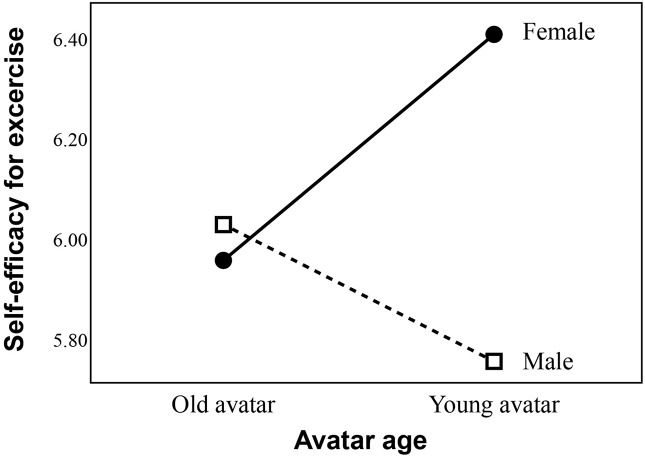
Interaction between avatar age and sex of participants in predicting self-efficacy for exercise.

Regarding self-efficacy regarding engagement in future exercise on the day of the experiment **(H1a and RQ3b)**, for participants who did not engage in vigorous activity, an interaction effect of avatar age and sex **(RQ3b)** was found (Figure 6), *F* = 4.88 (1, 100), *p* = 0.03, partial η^2^ = 0.05. The result of Levene’s test was not significant, *p* = 0.34. The simple effect analysis indicated that in the young avatar group, the female participants (*M* = 6.41, *SD* = 0.60, *N* = 27) reported significantly greater self-efficacy for exercise than the male participants (*M* = 6.41, *SD* = 0.60, *N* = 27), *p* = 0.01. Among the female participants, the young avatar group (*M* = 6.41, *SD* = 0.60, *N* = 27) also reported greater self-efficacy for exercise than the older avatar group (*M* = 5.96, *SD* = 0.96, *N* = 26), *p* = 0.05. The main effects of avatar age, *F* = 0.293 (1, 104), *p* = 0.59, partial η^2^ = 0.003, and sex, *F* = 3.19 (1, 109), *p* = 0.007, partial η^2^ = 0.03, were not significant. H1a was not supported. However, regarding RQ3b, the interaction effect on exercise self-efficacy was significant.

Regarding physical activity during the exercise phase (main effect, H1b and interaction effects, RQ3c), 2 × 2 ANOVAs were performed on the vector (overall magnitude regardless of direction) and on the step counts. When examining the distribution of the vector magnitude of physical activity, the outlier analysis through the boxplot identified three outliers in the data. Analyses including or excluding the outliers did not affect the results. However, for transparency, we report these separate analyses with and without outliers in Table 2. Here in the report we presented the analysis with the outliers. For the analysis with the outliers, regarding the vector magnitude of physical activity, no main effects were found: avatar age, *F* = 0.60 (1, 100), *p* = 0.44, partial η^2^ = 0.006; participant sex, *F* = 0.25 (1, 100), *p* = 0.62, partial η^2^ = 0.003. Regarding the main effect of avatar age on step counts, no significant main effects were found: avatar age, *F* = 2.18 (1, 100), *p* = 0.143, partial η^2^ = 0.021; participant sex, *F* = 3.66 (1, 100), *p* = 0.059, partial η^2^ = 0.035. Therefore, avatar age did not have the significant impact on physical activity; H1b was not supported.

**TABLE 2:** Statistics of the 2 × 2 ANOVAs for the vector magnitude of physical activity with and without outliers.

	With the outliers				Without the outliers			
	*F*(1, 100)	*p*	η^2^	Simple main effect	*F*(1, 97)	*p*	η^2^	Simple main effect
Avatar age	0.60	0.44	0.006		0.03	0.87	0.000	
Sex	0.25	0.62	0.003		0.75	0.39	0.008	
Avatar age × sex	6.57	0.01	0.062	Among Female: Young >Older * Young Avatar Group: Female > Male*	13.87	<0.001	0.125	Among Female: Young > Older *** Young Avatar Group: Female > Male ** Older Avatar Group: Female < Male *

*p < 0.05, **p < 0.01, ***p < 0.001.

**FIGURE 7:**
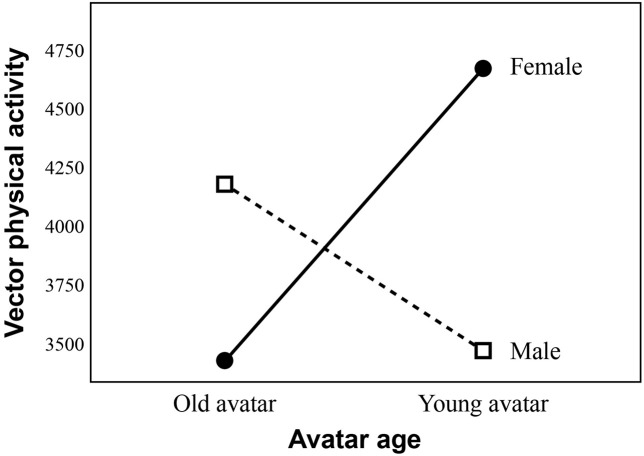
Interaction between avatar age and sex of participants in predicting vector physical activity.

For RQ3c, regarding the interaction effect on the vector magnitude of physical activity, the significant interaction effect of avatar age and sex was found for the vector of physical activity, *F* = 6.57 (1, 100), *p* = 0.012, partial η^2^ = 0.062. The result of Levene’s test was not significant, *p* = 0.90. To further explore the significant interaction effect (Figure 7, we presented the results with the outliers), we conducted a simple main effect analysis, which indicated that among the female participants, the young avatar group (*M* = 4657.88, *SD* = 1965.94, *N* = 27) demonstrated significantly greater vector physical activity than the older avatar group (*M* = 3416.91, *SD* = 2266.55, *N* = 26), *p* = 0.01. In addition, in the young avatar group, the female participants (*M* = 4657.88, *SD* = 1965.94, *N* = 27) had a significantly higher vector of physical activity than the male participants (*M* = 3519.22, *SD* = 1566.74, *N* = 26), *p* = 0.01. Regarding step counts, the interaction effects of avatar age and sex were not significant, *F* = 1.43 (1, 100), *p* = 0.234, partial η^2^ = 0.014.

To conclude, regarding physical activity, the significant interaction effect was found for the vector of physical activity. Young vs. older avatar embodiment had effects on the female participants’ physical activity. No main effects or interaction effect were found for step counts.

In addition to the exercise phase, we also explored the Proteus effect and sex differences in the above exercise outcomes during the voluntary phase and the next day. During the voluntary phase, no avatar effects were found on physical activities. Regarding next-day exercise self-efficacy and actual exercise, no significant effect was found for next-day exercise self-efficacy: avatar age, *F* = 1.31 (1, 100), *p* = 0.256, partial η^2^ = 0.013; participant sex, *F* = 0.202 (1, 100), *p* = 0.654, partial η^2^ = 0.002; interaction, *F* = 0.064 (1, 100), *p* = 0.83, partial η^2^ = 0.00. Regarding next-day actual exercise, the female participants reported significantly higher overall exercise, including vigorous, moderate, and light exercise time, than the male participants, *F* = 6.48 (1, 104), *p* = 0.012, partial η^2^ = 0.022.

No effects were found for self-concept (H2). For implicit self-concept, avatar age, *F* = 0.18 (1, 83), *p* = 0.670, partial η^2^ = 0.002; participant sex, *F* = 2.48 (1, 83), *p* = 0.119, partial η^2^ = 0.029; interaction, *F* = 0.12 (1, 83), *p* = 0.733, partial η^2^ = 0.001. Regarding explicit self-concept, avatar age, *F* = 1.16 (1, 100), *p* = 0.284, partial η^2^ = 0.011; participant sex, *F* = 3.60 (1, 100), *p* = 0.061, partial η^2^ = 0.035; interaction, *F* = 0.23 (1, 100), *p* = 0.630, partial η^2^ = 0.002. However, self-concept concerned whether the participants perceived themselves as young or old. In addition, the participants might have been affected by the arousing VR content, which may have affected their self-concept. Therefore, we controlled for age and perceived arousal as covariates of self-concept. After we controlled for age and perceived arousal, the main effect of sex on explicit self-concept was significant, *F* = 4.84 (1, 98), *p* = 0.03, partial η^2^ = 0.05. The female participants generally perceived themselves to be significantly younger (*M* = 3.87, *SD* = 1.39, *N* = 53; adjusted *M* = 3.854; *SE* = 0.19) than the male participants (*M* = 4.43, *SD* = 1.59, *N* = 51; adjusted *M* = 4.451; *SE* = 0.194). However, no such effects were found for implicit self-concept. H2 was not supported.

H3 and RQ2 explored whether self-concept mediated the relationship of avatar age with exercise outcomes. A series of bootstrapping analyses using the macro from Hayes (2017) shows that none of the mediating effects was significant (i.e., all confidence intervals include 0, indicating non-significance). H3 was not supported.

## Discussion

This study demonstrated that the Proteus effect through the manipulation of avatar age in VR is effective among elderly individuals in the context of exercise. The results showed that among elders who did not engage in vigorous exercise, the embodiment of younger avatars in VR leads to greater perceived exercise exertion, as male older adults with young avatars perceived themselves to contribute greater efforts to exercise. This finding shows that perceived exertion can have different interpretations; similarly, in Kocur et al. (2020), participants who embodied a muscular avatar perceived lower exertion than those embodying normal body avatars. In the present study, younger avatar age led to greater perceived exertion, which indicated that more efforts are devoted to aerobic exercise. Our conclusion of the Proteus effect is consistent with Kocur et al. (2020), in which different interpretations of perceived exertion as exercise outcome were observed. Future research and practitioners should predict different directions of perceived exertion based on various types of exercise.

This study found that sex moderated the effects of avatar age on self-efficacy for exercise and physical activity (i.e., vector magnitude). Greater Proteus effects on these parameters were observed for female than male elderly individuals. Female older adults who embodied young avatars reported greater self-efficacy regarding engagement in future exercise and greater physical activity (i.e., vector magnitude) during the exercise phase than those who embodied older avatars. This finding supports the assumption based on the previous literature that females more easily internalize thin or idealized bodies (Twamley and Davis, 1999; Murnen and College, 2019). This study suggests that females are more likely to be motivated to continue exercising through young avatar embodiment, indicating that young avatars are also a type of ideal body image. In addition, the female participants demonstrated that embodying young avatars motivated them to engage in more physical activity than the female participants who embodied older avatars. Among males, only a direct effect of young avatar embodiment on perceived exertion was observed, and no differences in other exercise outcomes were found. Our observations indicated that female participants were more willing to engage in various dimensions of the aerobic exercise moves, whereas male participants performed small-range moves. In the practice phase, the female participants were also more willing to try different exercise moves than male participants.

Moreover, older females who embodied old avatars reported significantly fewer vectors of physical activity than older males who embodied old avatars. This result indicated that females not only are prone to internalize the ideal body type into their physical activities but also are more easily affected by the Proteus effect of older avatars. The Proteus effect therefore was stronger and more significant among older females than among older males. Future studies should further examine the underlying mechanisms of such sex differences in VR embodiment.

This study also reported that perceived exertion is not significantly correlated with the vector magnitude of physical activity, implicitly suggesting that the psychological perception of efforts devoted to the exercise may not represent the counts of physical activities engaged in aerobic exercise. The evidence of the significant main effect of avatar age on the perceived exertion showed that the Proteus effect effectively led to psychological outcome mainly from subjective perception. The Proteus effect on physical activity is moderated by sex, which is significant only among female participants, suggesting the importance of sex regarding the physical outcomes. Indeed, one can engage in fewer physical activities but perceive that he/she had devoted great efforts to the exercise. Therefore, our study demonstrated that Proteus effect contributed to psychological outcomes (e.g., perceived exertion) regardless of sex and had conditional effect on physical outcomes moderated by sex. Our data showed that psychological and physical outcomes can be independent of the Proteus effect, which requires further investigation.

This study also found that female elderly individuals perceived themselves to be younger than male elderly individuals, and this trend persisted regardless of the type of avatar embodiment. Indeed, additional analysis showed that sex had an indirect effect on exercise self-efficacy through self-concept, with females having a younger self-concept, leading to their greater exercise self-efficacy. However, the moderated mediation model in which avatar embodiment was added as a moderator was not significant. Therefore, avatar age manipulation had a direct interaction effect with sex on exercise self-efficacy and physical activity but did not have an indirect interaction effect.

Although self-concept (Greenwald et al., 1998) was theorized to be the underlying mechanism of VR avatar embodiment on exercise outcomes, this study did not support this hypothesis. This might be due to the limitation of the timing of the measurement of self-concept. In this study, elderly individuals completed the IAT after they took off their VR goggles after the exercise and voluntary exercise phases. As character identification is a fleeting experience (Cohen, 2001), its measurement after the stimulus had ended may not have captured the participants’ temporarily altered self-concept. The participants were once again reminded of their age and actual bodies when completing the IAT. In addition, the null effect of the underlying mechanism might also have been due to the test itself. The IAT is a reaction association test, which seemed to be another task requiring cognition and hand-eye coordination for these elderly individuals. We also conducted additional analysis to explore body ownership (Slater, 2009) as a potential mechanism, and the data did not support this hypothesis either. Explicit self-concept was not a mediator either, but we found that after controlling for age and perceived arousal, the females perceived themselves as younger than the males. Future research should consider the sex difference in terms of the explicit self-concept of age. To address these limitations, future research could adopt simple self-concept measurements that ask participants to rate their self-perceived age during their exercise phase when they are embodying avatars.

This study also showed that Proteus effects were very short—they occurred only during the exercise phase and not during the voluntary phase and did not affect next-day self-efficacy and actual exercise. This finding implies that short-term avatar embodiment has a limited influence on exercise outcomes when participants are provided with instructions. This result is also consistent with a previous study (Reinhard et al., 2020) that found a Proteus effect in the first half of the experiment but not the second half. Nevertheless, during the experiment setting, the participants may not have felt free to continue to engage in more exercise during the voluntary phase. Intervention-based programs will be an ideal approach to further understand the Proteus effect among elderly individuals in future research.

This study is limited in some aspects. First, the short stimulus may not have allowed us to explore potential lasting effects on the next day. Merely reporting next-day exercise and next-day self-efficacy might not enable us to understand the potential effects of VR-based exercise. Nevertheless, the short exposure showed significant exercise outcomes through the manipulation of the age of the VR avatar. Future work can design a study covering a longer period to explore the duration, changes, and direction of effects to eliminate possible novelty effects of avatars on elders in exercise contexts. Second, the measurement approach for self-concept may not have fully represented the participants’ self-concept during VR embodiment. Furthermore, for elderly individuals’ safety, only simple exercise movements were designed in this study. Some participants noted that these movements were too easy for them to perform and that they would prefer more intense stimuli in the future. Future research could also compare the effects of young and old avatars on younger and older participants engaged in the same movements to explore the potential effects of avatar age on exercise. In addition, using several versions of young and old avatars could allow researchers to explore effects of visual differences and age on participants’ exercise. Despite some limitations, this study contributes to the literature as the first to provide empirical evidence for the Proteus effect among elderly individuals in the context of exercise. It also indicated that avatar age manipulation is an effective means to promote exercise among elderly individuals who do not engage in vigorous exercise and help them achieve exercise outcomes. The effect size obtained from this study is relatively moderate and higher than the average weighted r identified in Ratan’s meta-analysis regarding Proteus effects. Thus, manipulating elderly individuals’ age through avatars can have moderate effects on psychological and physical exercise outcomes. This study further demonstrated that female elderly individuals responded to young avatars differently than male elderly individuals, with female elderly individuals showing more positive effects of young avatar embodiment than males. These findings also have practical implications. Designers can focus on avatars’ age appearance and allow the manipulation of avatar age in exercise programs. Regarding sex differences, designers can allow male participants to customize their own avatars to best motivate them to exercise and increase their enjoyment. More research is needed to further examine the underlying mechanism and effective VR design for elderly individuals.

## Data Availability Statement

The datasets presented in this study can be found in online repositories. The names of the repository/repositories and accession number(s) can be found below: https://osf.io/63uw4/.

## Ethics Statement

The studies involving human participants were reviewed and approved by the National ChengChi University IRB. The patients/participants provided their written informed consent to participate in this study. Permission was obtained from the individual(s) for the use of any identifiable data or images included in this publication.

## Author Contributions

J-HT designed the study, wrote the entire manuscript, and worked on the revisions and proof. D-YW executed the entire experiment, including recruiting, executing, and follow-up, cleaned the data, prepared the data, and assisted in the method part during revisions. Both authors contributed to the article and approved the submitted version.

## Conflict of Interest

The authors declare that the research was conducted in the absence of any commercial or financial relationships that could be construed as a potential conflict of interest.

## Publisher’s Note

All claims expressed in this article are solely those of the authors and do not necessarily represent those of their affiliated organizations, or those of the publisher, the editors and the reviewers. Any product that may be evaluated in this article, or claim that may be made by its manufacturer, is not guaranteed or endorsed by the publisher.

## Footnotes

1
https://unity.com/

2
http://www.makehumancommunity.org/

3
https://actigraphcorp.com/support/software/actilife/
